# The Effects of Seed Pretreatment with Endophytic Bacteria *Bacillus subtilis* on the Water Balance of Spring and Winter Wheat Seedlings under Short-Time Water Deficit

**DOI:** 10.3390/plants12142684

**Published:** 2023-07-18

**Authors:** Alsu R. Lubyanova, Chulpan R. Allagulova, Oksana V. Lastochkina

**Affiliations:** Institute of Biochemistry and Genetics—Subdivision of the Ufa Federal Research Centre of the Russian Academy of Sciences, Prospect Oktyabrya 71, 450054 Ufa, Russia

**Keywords:** winter and spring *Triticum aestivum* L., drought stress, endophytic PGP bacteria, seed biopriming, osmotic potential, proline, relative water content, transpiration, water balance

## Abstract

We investigated the effect of pre-sowing seed treatment with endophytic *Bacillus subtilis* 10-4 (*B. subtilis*) on spring and winter wheat (*Triticum aestivum* L.; cultivars Ekada-70 (Ek) and Scepter (Sc), respectively) growth and tolerance under 1–24 h of drought stress, modulated by 12% polyethylene glycol 6000 (PEG). The results showed that drought decreased transpiration intensity (TI), root relative water content (RWC), osmotic potential (Ψ_π_) of cell sap, and induced proline accumulation and electrolyte leakage (EL) in both wheat cultivars. It was revealed that Sc was more responsive to PEG and *B. subtilis* treatments than Ek. Under drought, Ek did not significantly change root length, shoot height, or dry biomass. The pretreatment of wheat plants with *B. subtilis* performed significantly better under drought conditions through the enhanced TI, RWC, and Ψ_π_ of the cell sap in comparison with the plants treated with 12% PEG alone. *B. subtilis* also reduced stress-caused EL, especially in the Sc cultivar. Under water deficit wheat seedlings, pretreated with *B. subtilis*, have a higher proline accumulation in comparison to untreated stressed plants. Taken together, our results demonstrate the crucial role of endophytic *B. subtilis* in ameliorating the adverse effects of water stress on the water balance of both winter and spring wheat cultivars.

## 1. Introduction

Plants on Earth are exposed to different abiotic and biotic stress factors; drought is the most widespread among them [1,2,3]. Water deficit affects all parts of plant metabolism and causes a significant inhibition of the growth and productivity of cultivated plants [1,4]. Winter and spring bread wheat cultivars are the leaders among all grain crops in Russia and other countries worldwide. The cultivated areas of winter and spring wheat plants occupied more than 35% of the total crop area. Spring wheat seeds are sown in the spring, plants grow during the summer, and grains ripen in the fall [2]. Winter wheat seeds are sown in the fall and germinate before the cold weather; plants survive under the snow, continue their life cycle during the spring, and ripen earlier than spring crops. Spring cultivars grow more slowly and are more easily affected by weeds than winter cultivars. Winter cultivars tend to produce higher yields than spring cultivars due to their cold resistance and the ability to use the water obtained as a result of melting snow [2].

Drought is a serious problem that causes significant crop losses, affecting both spring and winter wheat cultivation areas. More than 60% of the world’s irrigated agricultural land is known to be affected by drought [3]. Water deficits reduce the growth, productivity, and grain quality of wheat [4] due to the decrease in transpiration rate, stomatal pore areas, relative water content, and water-use efficiency [5,6]. Drought is especially critical for wheat plants during the initial phases of development: germination and growth of young seedlings. Winter and spring wheat plants can have different reactions in terms of their water balance in changeable and stressful environmental conditions, in particular, under the influence of water deficit [2]. One of the key problems of modern biological science is the search for effective and environmentally safe ways to protect plants from adverse external influences.

Chemical fertilizers, fungicides, pesticides, and herbicides are widely used in crop production, but they have negative impacts on the environment and plants [7]. It is necessary to use safer agricultural techniques, and microbial-based natural plant biostimulants are an example of one of these approaches. There are a lot of strains of microorganisms in the natural environment [8,9] and the wheat rhizosphere as well [10]. The interactions between the microorganisms of plant rhizospheres are complex and may be negative [7,11,12], neutral, or beneficial on the basis of their effects on plant growth [13]. It is necessary to investigate all aspects of the life of plant growth-promoting (PGP) microorganisms, which provide benefits for plants due to the consortium effect and alleviate drought stress in crop plants [13,14], thereby improving growth and productivity [9,15,16,17,18,19,20]. Some bacterial isolates simultaneously are able not only to increase the growth of agricultural plants such as wheat, but are also able to decrease the growth of grass weed [7,21] and fungal diseases of wheat [7,22]. *Pseudomonas fluorescens* BRG100 produces substances with herbicidal activity on weed pest and could be used as bioherbicide [23]. The mechanisms of beneficial action of PGP bacteria and fungi for plant organisms involve releasing a wide range of important secondary metabolites: phytohormones [10,19,23,24,25,26] and organic and amino acids [12,24,25]. PGP bacteria could also enhance: photosynthesis [17,27,28,29], the nutrient uptake by solubilizing inorganic elements [7,25,28,29,30]; the biosynthesis of siderophores [10,13,16,20,25]; antioxidant defense systems [7,13,25,28,29]; and stress-responsive genes expression [13,15,16,19,26,31,32] under the influence of stress factors. PGP bacteria also induced the synthesis of phytohormones auxins, cytokinins, gibberellins, salicylic acid, and abscisic acid (ABA) in plants [14,25]. For example, PGP *Pseudomonas protegens* could change the ABA, cytokinin, and auxin contents in wheat plants, thereby increasing the size of the plant and its yield under drought stress [19]. It is known that several components of signaling pathways are common to both abiotic and biotic stimuli [33]. The interactions of Ca^2+^, reactive oxygen species, phytohormones, and downstream genes could lead to plant tolerance to adverse environmental stress factors. Worthy of particular attention is the isolation of drought-tolerant PGP bacterial strains from moisture-deficient conditions to increase plant drought tolerance [26]. Recent studies showed that drought-tolerant strains *Bacillus megaterium* MU2 and *B. licheniformis* MU8, isolated from the rhizosphere of plants of arid and semi-arid conditions, have caused systemic resistance of wheat to drought [26]. Drought-stressed wheat plants inoculated by drought-tolerant *Bacillus* spp. have a higher viability due to their increased relative water content, photosynthetic pigments, osmolytes (proline), and decreased lipid peroxidation [26,27].

*Bacillus subtilis* is one of the most attractive agents among PGP bacteria for the development of natural plant protection products, as it is generally recognized as safe for use in the food industry [10,34]. Moreover, *B. subtilis* produces endospores resistant to dynamic physical and chemical treatments, therefore maintaining their ability to trigger defense responses in host plants even under unfavorable conditions [34]. This makes it capable of the easy formulation and storage of *Bacillus*-based biological products. In recent times, it has been well-documented that *B. subtilis* interacts symbiotically or synergistically with host plants, representing a mutually helpful plant–bacteria interaction and possessing the ability to induce the growth and yield of plants under non-challenged growth and stress conditions [17,18,21,22,27,28,29,34]. Additionally, strains of *B. subtilis* are capable of colonizing the inner plant tissues (endophytes) considered to be more effective in plant stress protection than the rhizosphere strains (epiphytes) [34]. The beneficial effect of both the rhizosphere and endophytic *Bacillus* spp. is achieved by numerous direct and indirect similar mechanisms, including the production of a wide range of bioactive compounds, the regulation of plant hormones, photosynthesis, and decreasing stress-caused oxidative and osmotic damage [22,27,29,34,35]. The bacteria can also affect plant soil environments by reducing farmland soil nitrogen loss, improving nitrogen use efficiency and crop yield, reducing nitrification, and increasing denitrification [18,34,36]. These mechanisms of *Bacillus*-induced drought tolerance in plants were recently reviewed in detail [7,32,34]. However, many aspects of the interaction between endophytic *B. subtilis* and wheat under drought stress remain unclear and require further detailed investigation. 

Physiological, biochemical, and genetic plant responses to environmental stresses differ depending on plant cultivars [1,37,38,39,40]. The effectiveness of the same *B. subtilis* strain can vary depending on plant genotype, stresses, and other factors [27,34]. Wheat is a widely consumed crop, and the negative impact of drought on the water exchange of wheat plants is well documented [4,41,42,43]. However, the comparative analysis of the effects of endophytic bacteria *B. subtilis* on winter and spring wheat plants is limited [44], especially regarding water exchange during dehydration. The reaction of wheat plants to *B. subtilis* may also depend on their growing season under non-stressful and water-deficient conditions.

The aim of this work was to study the effect of pre-sowing seed treatment with endophytic bacteria *B. subtilis* 10-4 on the growth of spring and winter wheat seedlings, the integrity of their cellular structures, and the water metabolism parameters under normal growth conditions and water deficit, modulated by 12% polyethylene glycol 6000 (PEG).

## 2. Results

### 2.1. Growth Parameters

It was revealed that the root length and shoot height of winter wheat cultivar Scepter (Sc) were higher than those of spring wheat cultivar Ekada-70 (Ek) during non-stressed growth conditions (Table 1). Under normal growth conditions, the flag leaf area of Ek cultivar was higher than the leaf area of the Sc cultivar. *B. subtilis* 10-4 and 12% PEG application did not affect root length, shoot height, or flag leaf area of the Ek cultivar. The Sc cultivar turned out to be more sensitive to *B. subtilis* and 12% PEG treatments. Pretreatment with bacteria enhanced the root length by 13%, the shoot height—by 9%, and the flag leaf area—by 13% of the Sc cultivar in comparison to the control. Drought stress for 24 h decreased the Sc’s root length by 17%, the height of the flag leaf—by 9%, and the flag leaf area—by 13% in comparison to the control plants. Also, *B. subtilis* ameliorated the effect of 12% PEG on the linear size of the winter wheat Sc cultivar, while bacterial-treated wheat seedlings had the same root length, flag leaf height, and flag leaf area as control plants (Table 1).

The fresh weights (FW) of the Ek cultivar were higher than those of the Sc cultivar during normal (non-stressed) growth conditions (Table 2). *B. subtilis* inoculation increased the FW of both cultivars in comparison to the control, but a statistically significant enhancement of FW was observed in the roots of the Sc cultivar. Short-time water deficit exposure resulted in the decline of the FW of roots and shoots of both cultivars by 6–8% in comparison to control. Pretreatment with *B. subtilis* followed by the 12% PEG treatment for 24 h slightly increased FW (by 2–4%) in comparison to control non-bacterized and stressed wheat seedlings (Table 2).

The maximum values for dry weight (DW) were observed to be higher in the Ek cultivar than in the Sc cultivar under normal growth conditions (Table 3). *B. subtilis* increased the DW of roots and shoots of the Ek cultivar by 15% and 17%, respectively, in comparison to the control (Table 3) while, in the Sc cultivar, DW of roots and shoots increased upon *B. subtilis* treatment by 9% and 6%, respectively, in comparison to the control. A 12% PEG application for 24 h did not induce the changes in roots and shoots DW of the Ek cultivar, while the DW of roots and shoots of the Sc cultivar decreased by 4% and 6%, respectively, in comparison to control wheat seedlings. 

Under drought stress, in *B. subtilis*-pretreated winter wheat seedlings (Sc cultivar), the DW of roots increased by 6% and that of shoots—by 4% in comparison to untreated and stressed plants. As for spring wheat (Ek cultivar), a water deficit for 24 h did not change the DW of bacterized wheat plants in comparison to seedlings pretreated with *B. subtilis* alone (Table 3).

### 2.2. Transpiration Intensity (TI)

**Figure 1:**
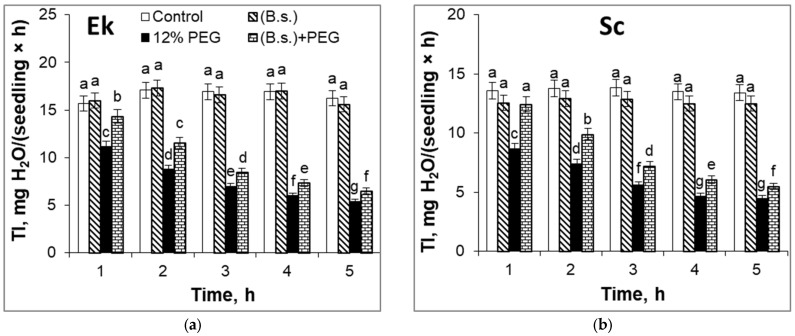
The effect of pre-sowing seed treatment with *B. subtilis* 10-4 (B.s.) of spring wheat cultivar Ekada-70 (Ek, (**a**)) and winter wheat cultivar Scepter (Sc, (**b**)) on the transpiration intensity (TI) of leaves under water deficit, modulated by 12% PEG for 5 h. Data are the mean of three replicates; different letters show significant differences at *p* ≤ 0.05, and bars indicate ± SE (*n* = 20).

The leaves of spring wheat (Ek cultivar) (Figure 1a) demonstrated a higher transpiration intensity (TI) than winter wheat (Sc cultivar) (Figure 1b) during non-challenged growth conditions. *B. subtilis* pretreatment did not affect the TI of the Ek shoots but reduced the TI of the Sc shoots by 6–8% in comparison to control plants (Figure 1). Under the first hour of drought stress exposure, modulated by 12% PEG, a decrease in TI in shoots of Ek (by 29%) and Sc (by 36%) cultivars was observed. However, a difference in TI was not observed during further stress application. Figure 1 shows that a further water deficit provoked a decrease in TI equally in the leaves of both spring and winter wheat cultivars. Application of *B. subtilis* increased the TI of the leaves of 7-day-old seedlings in comparison to water deficit stress alone. The findings showed that pretreatment with *B. subtilis* has an especially beneficial effect on the TI parameter at the beginning of stress exposure (Figure 1). Under the first hour of drought stress, *B. subtilis* pretreatment reduced the TI by only 9% in the Ek cultivar (Figure 1a) and by 8% in the Sc cultivar (Figure 1b) in comparison to the control. 

### 2.3. Relative Water Content (RWC)

**Figure 2:**
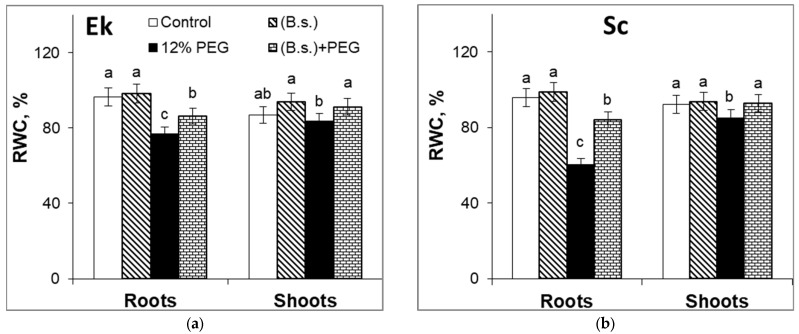
The effect of seed inoculation with *B. subtilis* 10-4 (B.s.) on relative water content (RWC) in roots and shoots of spring wheat cultivar Ekada-70 (Ek, (**a**)) and winter wheat cultivar Scepter (Sc, (**b**)) exposed to 12% PEG for 5 h. Data are the mean of three replicates; different letters show significant difference at *p* ≤ 0.05 and bars indicate ± SE (*n* = 15).

Figure 2 illustrates that *B. subtilis* did not change the RWC of the roots of the Ek cultivar or the roots and shoots of the Sc cultivar in comparison to the control. RWC levels increased by 7% in shoots of the Ek cultivar pretreated with this PGP bacteria in comparison to the control (Figure 2a). It should be noted that the control shoots’ RWC level of winter wheat plants was higher (by 6%) than that of spring wheat plants. 

Drought stress exposure for 5 h resulted in reduced root RWC in Ek (by 22%) (Figure 2a) and Sc (by 35%) (Figure 2b) cultivars in comparison to control. On the other hand, water stress did not affect the shoot RWC level of seedlings of both wheat cultivars significantly and decreased shoot RWC in Ek (by 3%) and Sc (by 7%) in comparison to control plants. *B. subtilis* alleviated the damage effect of drought on water content by increasing the RWC levels. Pretreatment with *B. subtilis* and subsequent 12% PEG application induced an increase in RWC levels in the roots of Ek (by 7%) and Sc (by 17.5%) cultivars in comparison to the sole PEG application. In *B. subtilis*-pretreated and stressed plants, shoot RWC increased by 7.6–7.8% in comparison to stressed plants alone (Figure 2).

### 2.4. Osmotic Potential (Ψ_π_) of Wheat Sap

**Figure 3:**
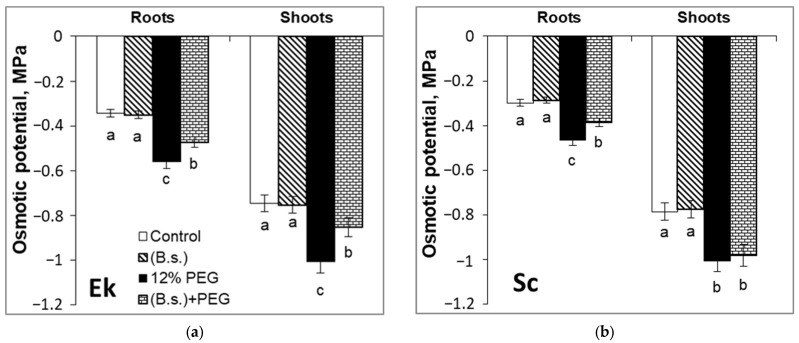
The effects of pre-sowing seed treatment with *B. subtilis* 10-4 (B.s.) of spring wheat of Ekada-70 cultivar (Ek, (**a**)) and winter wheat of Scepter cultivar (Sc, (**b**)) on the osmotic potential of roots and shoots sap under drought stress, modulated by 12% PEG. Data are the mean of three replicates; different letters show significant differences at *p* ≤ 0.05 and bars indicate ± SE (*n* = 30).

It was revealed that *B. subtilis* did not affect osmotic potential parameters (Ψ_π_) of both spring and winter wheat cultivars (Figure 3). Bacterized non-stressed seedlings had the same Ψ_π_ of shoots and roots as the control ones. A 12% PEG application for 24 h caused a decrease in root Ψ_π_ in Ek (by 63%) (Figure 3a) and Sc (by 56%) (Figure 3b) plants as compared to control seedlings. Under stress, shoot Ψ_π_ dropped by 35% in Ek and 28% in Sc cultivars compared with the control groups. The osmotic potentials of the roots and the shoots of *B. subtilis*-inoculated Ek plants were increased during drought exposure. Particularly, *B. subtilis* increased the Ψ_π_ parameter in roots by 16% and shoots by 14% of Ek seedlings under 12% PEG compared to non-bacterized and stressed plants (Figure 3a). Also, *B. subtilis* increased the root Ψ_π_ of winter wheat (Sc) by 29% but did not affect the shoot Ψ_π_ in comparison to non-bacterized and stressed plants (Figure 3b). 

### 2.5. Proline Content

**Figure 4:**
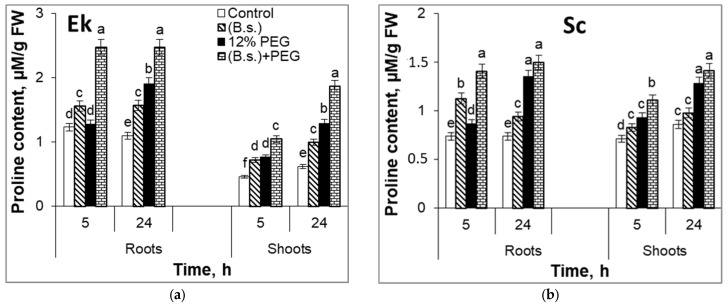
Proline content in roots and shoots of 7-day-old seedlings of spring and winter wheat cultivars Ekada-70 (Ek, (**a**)) and Scepter (Sc, (**b**)), respectively, inoculated with *B. subtilis* 10-4 (B.s.) and exposed to 12% PEG for 24 h. Data are the mean of three replicates; different letters show significant differences at *p* ≤ 0.05 and bars indicate ± SE (*n* = 15).

It was revealed that the roots of spring wheat (Ek cultivar) contained 1.7-fold more proline than the roots of winter wheat (Sc cultivar) under non-stressed growth conditions (Figure 4). As for shoots, the concentration of this amino acid in the Sc cultivar was about 1.7-fold higher in comparison to the Ek cultivar under normal conditions. The proline content in non-stressed roots and shoots of the Sc cultivar did not differ (Figure 4a), but Ek cultivar roots had a 1.8–2.7-fold increase in production of proline in roots than in shoots (Figure 4b). *B. subtilis* induced an increase in proline accumulation in shoots more than in roots. Figure 4 illustrates that bacterial treatment enhanced proline accumulation in spring wheat (Ek) roots (by 27–43%) and shoots (by 57–61%) in comparison to control (Figure 4a). The contents of proline in *B. subtilis*-pretreated winter wheat (Sc) roots and shoots were increased by 27–51% and 14–17%, respectively, in comparison to control (Figure 4b).

Drought resulted in an increased proline content in Ek cultivar roots (by 4–74%) and shoots (by 66–108%) relative to non-challenged growth conditions (Figure 4a). A 12% PEG induced proline accumulation in roots (by 18–27%) and in shoots (by 14–31%) of Sc cultivar as well (Figure 4b). The results of our experiments indicated that endophytic bacteria *B. subtilis* could induce the additional drought-induced proline increment under the initial stage of water-limiting conditions. Moreover, during water deficit (for 24 h), the *B. subtilis*-pretreated Ek cultivar displayed greater accumulation of this amino acid than the *B. subtilis*-pretreated Sc cultivar (Figure 4). In particular, under 12% PEG application for 24 h, the bacterized seedlings increased proline accumulation in Ek cultivar’ roots by 29–93% and shoots by 36–45% in comparison to non-bacterized and stressed plants (Figure 4a), while the content of proline in bacterized Sc cultivar was increased in roots by 11–62% and in shoots by 10–19% in comparison to non-bacterized plants under water deficit (Figure 4b). 

### 2.6. Electrolyte Leakage (EL)

**Figure 5:**
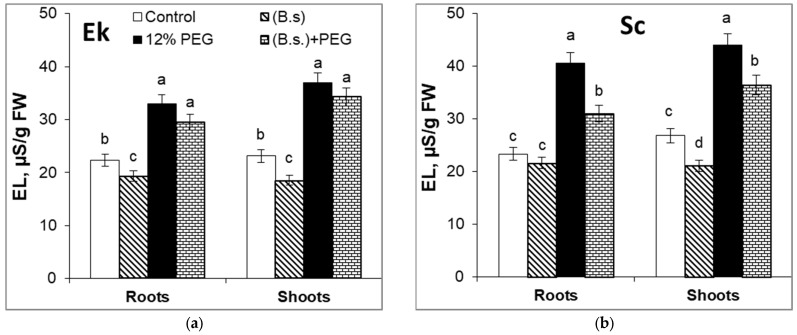
The effects of *B. subtilis* 10-4 (B.s.) pre-sowing seed inoculation and 12% PEG application for 24 h on electrolyte leakage (EL) of roots and shoots of spring wheat cultivar Ekada-70 (Ek, (**a**)) and winter wheat cultivar Scepter (Sc, (**b**)). Data are the mean of three replicates; different letters show significant differences at *p* ≤ 0.05 and bars indicate ± SE (*n* = 20).

The results showed that, in the presence of *B. subtilis* 10-4, the EL of the Ek cultivar (spring wheat) was reduced in roots by 13% and in shoots by 20% in comparison to the control (Figure 5a). As for the Sc cultivar (winter wheat), *B. subtilis* inoculation decreased the EL of roots by 8% and that of shoots by 21% in comparison to control plants (Figure 5b). After 24 h of 12% PEG application, EL was higher in the Sc cultivar than in the Ek cultivar (Figure 5). 

Water deficit enhanced the EL of the Ek cultivar by 48% in roots and by 60% in shoots in comparison to the control (Figure 5a) while, in Sc cultivar, stress increased EL by 74% in roots and 64% in shoots as compared to control seedlings (Figure 5b). It was revealed that in *B. subtilis*-pretreated and stressed wheat plants, EL was lower than in stressed seedlings alone. Moreover, during a water deficit, *B. subtilis* statistically significantly reduced EL in the Sc cultivar than in the Ek cultivar (Figure 5). In particular, under drought stress, *B. subtilis* pretreatment of the Ek cultivar led to EL values that were enhanced in roots by 32% and in shoots by 48% over the control (Figure 5a). During a water deficit, *B. subtilis* enhanced the EL of the Sc cultivar by 33% (in roots) and by 36% (in shoots) in comparison to the control (Figure 5b). 

## 3. Discussion

It is known that drought reduces the growth parameters of plants [43], and the presence of certain endophytic PGP bacteria can enhance seed germination percentage and stimulate plant growth [5]. These PGP microbes have been found to improve the growth parameters of various plants, including *Achnatherum inebrians* [45], tomato [46], common bean and maize [47], black gram and green pea [13], Chinese cabbage [18], soybean [16], and wheat [19,27,34,42,48]. The inoculation of crop seeds with PGP microbes has shown potential in stimulating plant growth under different environmental conditions [43], including abiotic stresses and normal growth conditions. Additionally, PGP microbes have been found to enhance the vigor of grass *Achnatherum inebrians* seeds stored under low and intermediate moisture levels [45].

The mechanisms of the regulatory activity of PGP microbes on plant growth and physiology are actively investigated. The indirect promotion of plant growth occurs when PGP microbes mitigate the deleterious effects of phytopathogenic organisms by producing antagonistic substances or by inducing resistance to pathogens [14,34]. Direct microbial-induced plant growth promotion happens by modulating the phytohormonal balance of plants by producing phytohormones or facilitating the uptake of certain nutrients from the environment. It is known that *B. subtilis* stimulates seed and plant growth due to aminocyclopropane-1-carboxylate (ACC) deaminase production [22,46], thereby reducing stress-induced ethylene production and leading to plant growth promotion [11]. ABA is also involved in plant water metabolism regulation during drought [23]. Undoubtedly, PGP microbes may interfere with and cross-talk with phytohormones signaling networks [14]. Various trends of ABA accumulation in shoots and roots of wheat under the influence of PGP microbes, for example, *B. subtilis* IB-22 and *Pseudomonas mandelii* IB-Ki14, could regulate the water balance of plants under salinity [48]. It was shown that *B. subtilis* increased root growth, tillering, stalk weight, and sucrose concentration in the stalks of sugarcane due to improved water use efficiency under drought stress [28]. The increase in leaf area of tomato plants by microalgae-cyanobacteria extract treatment was due to improved osmotic adjustments and ion homeostasis [49]. 

The plant growth stimulating effect of bacteria depends on its strain and the host plant [13,47]. Drought-induced reduction in DW in chickpea plants depended on their stress-sensitivity [12]. But there is limited information on the comparative analysis of the PGP microbes influence on spring and winter wheat cultivars under water deficit conditions [2]. In our experiments, a difference in the growth responses of spring wheat and winter wheat varieties to bacterial treatment and osmotic stress was revealed. During the first seven days from germination, the winter wheat plants of the Sc cultivar grew rapidly in length, with less water uptake, accumulation of dry substances, and deployment of the leaf blade under non-challenged growth conditions in comparison to the spring wheat plants (Ek cultivar) (Table 1, Table 2 and Table 3). The strategy of growth of spring wheat of the Ek cultivar during the first days after germination is the enhancement of the leaf area and water and dry substance accumulation. The bacterial inoculation of Ek plants had no significant effect on their linear sizes (Table 1) or FW (Table 2), but stimulated root and shoot DW (Table 3) in comparison to the control. The winter wheat of the Sc cultivar also showed sensitivity to *B. subtilis* 10-4 treatment. Bacterial pretreatment significantly increased the length and FW of roots, flag leaf area, and DW of shoots in comparison to the control (Table 1, Table 2 and Table 3). The initial stage of drought influenced plants’ growth and biomass accumulation, and this is especially reflected in the reduced root length of the Sc cultivar (Table 1). At the same time, the DW of stressed spring wheat of the Ek cultivar was the same as that of control plants (Table 3). A water deficit reduced the flag leaf area of the Ek cultivar, but it was statistically insignificant in comparison to the control plants. The improvement in the growth parameters of *B. subtilis*-inoculated plants was especially noticeable in the winter wheat (Sc cultivar) during drought. 

The regulation of the water status of crop plants is vital to confer drought stress tolerance. One of the first water-deficit-induced physiological responses of plants is the reduction in the stomata opening [4,6,43], and transpiration intensity is directly related to this process. During the first hour of 12% PEG application, the TI of the Sc cultivar (winter wheat) decreased by 7% more than the TI of the Ek cultivar (spring wheat). The further reduction in TI was comparable in both cultivars. Under water deficit, the bacterized wheat plants of both cultivars maintained increased TI in comparison to non-bacterized and stressed seedlings (Figure 1). Interestingly, the TI of the winter wheat (Sc cultivar) was slightly reduced even upon pretreatment with bacteria. It is known that PGP microbes could regulate the transpiration process. For instance, *B. firmus* SW5 inoculation significantly boosted the transpiration rate, net photosynthesis rate, and stomatal conductance of soybean plants under control and salt-stressed conditions [15]. *B. thuringiensis* AZP2 could increase the water use efficiency of wheat plants [44].

*B. subtilis* did not change RWC levels of the roots of the Ek cultivar or the roots and shoots of the Sc cultivar, but promoted slight water accumulation in shoots of the Ek cultivar (Figure 2) by maintaining TI and activating biosynthesis (Figure 3 and Figure 4), contributing to the water accumulation. It is known that *B. subtilis* was able to increase the water content of Chinese cabbage [16] under non-stressful conditions. Drought stress is also capable of decreasing the plant’s RWC owing to the water deficit in the environment [6]. The presence of 12% PEG in the nutrient solution negatively regulated plant water accumulation, in particular in roots, which was the site of stress influence (Figure 2). The RWC level of spring wheat (Ek cultivar) decreased less than that RWC of winter wheat (Sc cultivar). The pre-sowing seed treatment with *B. subtilis* influenced the RWC level of wheat seedling of both cultivars and promoted the increase in water accumulation under drought stress. The bacterization helped to increase the RWC level of the roots of the Sc cultivar to a greater extent than the roots of the Ek cultivar. Data in the literature showed that *B. subtilis* increased the RWC of tomato plants [46], common beans, and maize [47] under normal and drought conditions. The inoculation with PGP microbes increased leaf water content and influenced the TI due to the regulation of stomata. *P. protegens* could contribute to the normalization of water uptake by wheat plants under arid conditions [19]. Under osmotic stress, PGP microbes increased the RWC of black gram and green pea in comparison to non-bacterized and stressed plants [13]. It is known that PGP microbes significantly reduced the RWC damage in both drought-sensitive and drought-tolerant varieties of *Cicer arietinum* grown under stress conditions [50]. The bacterial treatment better enhanced the RWC of the drought-sensitive variety compared to the tolerant variety. 

The accumulation of osmotically active substances in plant cell sap and the reduction in osmotic potential are general components of plant adaptation during water deficit (Figure 3) [4,6,43]. The effect of *B. subtilis* pretreatment on Ψ_π_ was not observed in wheat seedlings of both cultivars under non-stressful conditions. The higher Ψ_π_ level in the roots and shoots of the Ek cultivar (spring wheat), and in the roots, but not the shoots, of the Sc cultivar (winter wheat) was a result of endophyte presence during water stress. The protective effect of PGP bacteria is related to its influence on the accumulation of osmotic substances in plants under water deficit. *B. subtilis*-pretreated wheat seedlings, especially Ek cultivar (spring wheat), needed to synthesize and accumulate less osmotics than Sc cultivar (winter wheat) and direct resources to other important physiological and biochemical processes.

Proline accumulation in plant tissues during environmental stress is correlated with stress tolerance [4,51,52]. This amino acid may act as a signaling molecule, an osmoprotectant, metal chelator, or a participant in the oxidative stress defense system [53,54,55]. Our results demonstrated that spring wheat (Ek cultivar) accumulates proline more in the roots than in the shoots (Figure 4). The proline content was lower in the shoots of spring wheat (Ek cultivar) than in winter wheat (Sc cultivar). Proline accumulation was significantly enhanced by *B. subtilis* pretreatment in both wheat cultivars. These results are supported by data in the literature: plant growth-stimulating microorganisms induced proline accumulation in grass seeds [45], soybean [16], tomato [46], black gram, and green pea [13]. Despite the fact that Fonseca et al. (2022) showed that *B. subtilis* could reduce proline accumulation during stress, our results (Figure 4) and data from the literature [13,16,46,49,50] demonstrated PGP microbe-induced additional proline accumulation in plants under environmental stresses.

Data in the literature [37,52,56] and our results (Figure 5) confirm that environmental stresses lead to membrane disruption and a significant increase in EL in comparison to the control. The winter wheat seedlings (Sc cultivar) obviously respond better to osmotic stress and water deficits at the early stage of plant development than the spring wheat (Ek cultivar). *B. subtilis*-pretreated wheat seedlings of the Sc cultivar had lower EL than unpretreated and stressed plants, which shows the increased membrane resistance to drought stress. PGP microbes did not reduce stress-induced EL in the Ek cultivar to the same extent as in the Sc cultivar. The EL parameters of *B. subtilis*-inoculated and stressed plants of both cultivars were nearly the same. It is known that endophyte *Epichloe* was able to decrease the electrical conductivity of grass seeds as compared to control in almost all moisture contents [45].

The interest of researchers is directed to the evaluation of wheat plants with different tolerances to drought in terms of physiological, biochemical, and genetic responses [43], but plant stress responses of spring and winter plants are discussed separately [4,5,6,21,43,57,58]. There are limited works concerning the comparative analysis of winter and spring plants under abiotic stresses and the effects of endophytic bacteria on these plants [59,60]. We received the priority data that pre-sowing incubation of wheat seeds in *B. subtilis* 10-4 suspension culture for 1 h has a positive effect on the water balance of both spring and winter wheat seedlings. The strategy of spring wheat (Ek cultivar) was the induction of biosynthesis, which affects its DW, proline, and osmotic potential levels during normal and stressful growth conditions. The winter wheat (Sc cultivar) stimulates more water uptake, which reflects the FW, length parameters, TI, and RWC. 

## 4. Materials and Methods

### 4.1. Plant Material, Bacterial Strain, and Inoculum Preparation

The seeds (*Triticum aestivum* L.) of winter wheat cultivar Scepter (Sc) and spring wheat cultivar Ekada-70 (Ek) were obtained from Chishminsky Breeding Station UFRC RAS (Chishmy, Ufa, Russia).

Endophytic PGP bacteria *B. subtilis* strain 10-4 was earlier isolated from the dryland arable soils of the Republic of Bashkortostan (Russia). Based on the results of the sequencing analysis of the variable regions of genes encoding 16S rRNA as well as PCR analysis using species-specific primers (secYsubF TTATATCACGGCTTCGAT, secYsubR CGGTAGTTTCGTTTCACCA), the strain 10-4 was identified as *B. subtilis* (99%) in our previous work [61] and deposited in the All-Russian Collection of Industrial Microorganisms (number B-12988). On solid Luria-Bertani (LB) nutrient medium, the colonies of strain 10-4 are round with a wavy edge, beige-white in color, and have a smooth surface that is rough in the center. The profile of the colony is flat; the colonies are matte with a slight glitter in the center; the texture is semi-dry; the consistency is soft, slightly mucous. Strain 10-4 is drought-tolerant [27], capable of producing IAA and siderophores, fixing atmospheric nitrogen, and colonizing internal wheat tissues (endophyte) [42,61]. 

To obtain inoculum, *B. subtilis* 10-4 cells were cultured in liquid LB nutrient medium at 180 rpm, 37 °C for 24 h. For pre-sowing seed inoculation, the bacterial culture was used at a concentration of 10^5^ colony-forming units (CFU) mL^−1^ [61].

### 4.2. Seed Treatment, Experimental Design, and Growth Conditions

The seeds were surface sterilized with 96% ethanol, washed with water, air-dried, incubated in a suspension of *B. subtilis* 10-4 (10^5^ CFU mL^−1^) or water (control) for 1 h [42], and sown in Petri plates with filter paper moistened with water. Thereafter, 2-day-old seedlings pretreated and untreated with bacteria were grown in glass jars on a 10% Hoagland–Arnon solution for 4 d (16-h photoperiod, illumination of 200 mmoL m^−2^s^−1^ and temperature of 22–23 °C). Changing the solution every day was sufficient. The degree of drought tolerance strongly depends on the developmental stage of most plant species, so we used the sensitive first leaf stage of wheat plants in our investigation. Then, 6-day-old plants were subjected to the short-time water deficit induced by 1–24 h of 12% PEG (PanReac AppliChem, Barcelona, Spain) application prepared in a 10% Hoagland–Arnon solution. This osmotic stress reduces the ability of plants to take up water, thus modulating drought. The remaining wheat plants continued to grow on a 10% Hoagland–Arnon solution. Furthermore, 6- or 7-day-old seedlings were analyzed for different root and shoot parameters, including growth, transpiration intensity (TI), relative water content (RWC), osmotic potential of cell sap (Ψ_π_), electrolyte leakage (EL), and proline accumulation.

### 4.3. Measurement of the Relative Water Content (RWC)

The fresh weight was determined immediately after separating the leaves and roots from the plant. To determine turgor weight, shoots were placed at room temperature for 24 h in the dark in closed vessels, loading the base into distilled water [62], while roots were completely immersed in water for the same period [63]. Then, samples were dried in open containers, which provided free circulation of air, in a hot air oven at 70 °C until a constant dry mass was achieved. RWC was estimated by following the formula:

RWC = [(Fresh weight − Dry weight)/(Turgid weight − Dry weight)] × 100%.

### 4.4. Estimation of the Transpiration Intensity

Transpiration expressed per seedling was measured gravimetrically for 10 plants per treatment. To prevent evaporation of the 10% Hoagland–Arnon solution from the surface of the beakers, they were covered with aluminum foil. A small slit for the shoot was made on the top of the beaker to allow the plant to grow through. The initial and final (after each time point) beaker weights were taken, and transpiration was calculated by subtracting the final beaker weight from the initial weight divided by the number of seedlings [64].

### 4.5. Osmotic Potential Estimation

Wheat roots and shoots (6 days old) were fixed at −20 °C, then thawed and the released sap centrifuged. The osmotic potential of cell sap was measured using a digital microosmometer (Camlab Ltd., Cambridge, UK) [41].

### 4.6. Proline Accumulation

For proline determination, 0.5 g of fresh shoot and root material were soaked in 3 mL of hot distilled water and boiled in a water bath for 1 h at 100 °C [65]. The immediately cooled extract (1 mL) was then mixed with acid-ninhydrin reagent (1 mL) and glacial acetic acid (1 mL) in a test tube, and the mixture was placed in a water bath for 1 h at 100 °C. The absorbance of the cold reaction mixture was measured at 522 nm using a Smart Spec Plus spectrophotometer (Bio-Rad, Hercules, CA, USA). The proline concentration in each wheat tissue sample was calculated by comparison with a standard curve of proline.

### 4.7. Determination of the Leaf Area

Leaf area was measured using a planar scanner and ImageJ software (National Institutes of Health, Bethesda, MD, USA).

### 4.8. Determination of the Electrolyte Leakage (EL)

The roots and shoots of wheat seedlings were rinsed with distilled water after all treatments and incubated in a glass jars containing 20 mL of distilled water, stirring on an ES-20 shaker (Biosan, Riga, Latvia) at 130 rpm, 30 °C for 2 h. The electrical conductivity of the incubation medium was determined by a portable multi-band conductometer/TDS meter HI 8633 (HANNA Instruments, Catania, Italy) [37]. The results are given as the electrical conductivity µS/g FW.

### 4.9. Statistical Analysis

All experiments were performed with three biological replications and three technical repetitions each. The data presented were mean values with standard errors (±SE). Statistical data processing was carried out using a statistical analysis package in Microsoft Office Excel 2010. Means were compared using analysis of variance (ANOVA) with *p* ≤ 0.05.

## 5. Conclusions

This article has highlighted the significant role that endophytic bacteria *B. subtilis* 10-4 can play in promoting spring and winter wheat growth and water acquisition improvement. The findings showed that the 12% PEG-induced changes in the levels of growth, transpiration intensity, relative water content, osmotic potential of cell sap, electrolyte leakage, and proline accumulation parameters were partially reversed by pre-sowing seed treatment with this bacterium. Pretreatment with *B. subtilis* 10-4 positively regulated various water balance parameters of vulnerable young wheat seedlings of both spring and winter wheat under drought stress, thereby imparting stress tolerance during water deficit. Thus, *B. subtilis* 10-4 has the potential to be used as an eco-friendly approach for spring and winter wheat production for growth stimulation and yield preservation under unfavorable water deficit conditions.

## Figures and Tables

**Table 1:** The effect of pre-sowing seed treatment with *B. subtilis* 10-4 and 12% PEG application for 24 h on growth parameters of 7-day-old spring (Ek) and winter (Sc) wheat plants.

Cultivar	Treatment	Root Length, cm	Shoot Height, cm	Flag Leaf Area, cm^2^
Ek	Control	8.7 ± 1.1	14.6 ± 1.2	3.5 ± 0.30
	(B.s.)	8.6 ± 1.1 ^ns^	14.4 ± 1.0 ^ns^	3.6 ± 0.30 ^ns^
	12% PEG	8.4 ± 1.7 ^ns^	14.2 ± 0.8 ^ns^	3.2 ± 0.16 ^ns^
	(B.s.) + PEG	8.8 ± 1.5 ^ns^	14.2 ± 1.1 ^ns^	3.4 ± 0.25 ^ns^
Sc	Control	11.1 ± 1.7	16.1 ± 1.2	3.2 ± 0.35
	(B.s.)	12.5 ± 1.8 *	17.5 ± 1.3 ^ns^	3.6 ± 0.30 *
	12% PEG	9.2 ± 1.4 *	15.9 ± 1.5 ^ns^	3.1 ± 0.35 ^ns^
	(B.s.) + PEG	11.6 ± 1.2 ^ns^	16.7 ± 1.1 ^ns^	3.3 ± 0.30 ^ns^

(B.s.)—*B. subtilis* 10-4 pretreatment; PEG—polyethylene glycol 6000; Ek—Ekada-70 cultivar; Sc—Scepter cultivar. The data are mean ± SE (*n* = 30). * indicates significant difference and ^ns^ indicates non-significant difference at *p* ≤ 0.05 between control and treatments.

**Table 2:** The effect of pre-sowing seed treatment with *B. subtilis* 10-4 (B.s.) and 12% PEG application for 24 h on the fresh weight (FW) of 7-day-old spring (Ek) and winter (Sc) wheat plants.

Cultivar	Treatment	Root Weight, mg	Shoot Weight, mg
Ek	Control	56.9 ± 2.8	100.7 ± 5.0
	(B.s.)	58.6 ± 3.0 ^ns^	109.2 ± 5.4 ^ns^
	12% PEG	52.6 ± 2.6 ^ns^	94.8 ± 4.7 ^ns^
	(B.s.) + PEG	53.8 ± 2.7 ^ns^	97.7 ± 4.9 ^ns^
Sc	Control	54.0 ± 2.7	95.5 ± 4.7
	(B.s.)	65.3 ± 3.2 *	100.9 ± 5.0 ^ns^
	12% PEG	50.5 ± 2.5 ^ns^	89.3 ± 4.4 ^ns^
	(B.s.) + PEG	52.5 ± 2.6 ^ns^	90.7 ± 4.5 ^ns^

The data are mean ± SE (*n* = 30). * indicates a significant difference, and ^ns^ indicates a non-significant difference at *p* ≤ 0.05 between control and treatments.

**Table 3:** The effect of pre-sowing seed treatment with *B. subtilis* 10-4 (B.s.) and 12% PEG application for 24 h on the dry weight (DW) of 7-day-old spring (Ek) and winter (Sc) wheat plants.

Cultivar	Treatment	Root Weight, mg	Shoot Weight, mg
Ek	Control	4.82 ± 0.24	9.74 ± 0.47
	(B.s.)	5.61 ± 0.28 *	11.35 ± 0.56 *
	12% PEG	4.85 ± 0.24 ^ns^	9.91 ± 0.50 ^ns^
	(B.s.) + PEG	5.52 ± 0.28 *	11.29 ± 0.56 *
Sc	Control	4.17 ± 0.20	9.24 ± 0.49
	(B.s.)	4.53 ± 0.23 ^ns^	10.40 ± 0.52 *
	12% PEG	4.01 ± 0.20 ^ns^	9.18 ± 0.46 ^ns^
	(B.s.) + PEG	4.27 ± 0.21 ^ns^	9.52 ± 0.48 ^ns^

The data are mean ± SE (*n* = 30). * indicates a significant difference, and ^ns^ indicates a non-significant difference at *p* ≤ 0.05 between control and treatments.

## Data Availability

Not applicable.
